# Hormone receptors AR, ER, PR and growth factor receptor Her-2 expression in oral squamous cell carcinoma: Correlation with overall survival, disease-free survival and 10-year survival in a high-risk population

**DOI:** 10.1371/journal.pone.0267300

**Published:** 2022-05-11

**Authors:** Yumna Adnan, Syed Muhammad Adnan Ali, Muhammad Sohail Awan, Romana Idress, Muhammad Ozair Awan, Hasnain Ahmed Farooqui, Hammad Afzal Kayani

**Affiliations:** 1 Office of Academia and Research in Surgery, Department of Surgery, Aga Khan University Hospital, Karachi, Pakistan; 2 Department of Biosciences, Faculty of Life Sciences, Shaheed Zulfikar Ali Bhutto Institute of Science and Technology, Karachi, Pakistan; 3 Section of Otolaryngology–Head and Neck Surgery, Department of Surgery, Aga Khan University Hospital, Karachi, Pakistan; 4 Section of Histopathology, Department of Pathology and Laboratory Medicines, Aga Khan University Hospital, Karachi, Pakistan; 5 Medical College, Aga Khan University Hospital, Karachi, Pakistan; Turun Yliopisto, FINLAND

## Abstract

Oral squamous cell carcinoma (OSCC) comprises most of head and neck neoplasms and is one of the highest-ranking and lethal cancers in Pakistan due to prevailing mouth habits. Several types of receptors act as prognostic markers and targets for therapy in some cancers, but their application in OSCC is largely unexplored. This study aimed to evaluate the expression of hormonal receptors and Her-2 in OSCC patients and correlate it with 10-year, overall and disease-free survival. To achieve this objective, immunohistochemistry for Her-2, AR, ER and PR was performed on 100 formalin-fixed paraffin-embedded primary OSCC specimens. Receptor expression was correlated with mouth habits and clinicopathological features and patient survival was analyzed using Kaplan-Meier method and Cox regression univariate analysis. We observed that in 100 patients, there were 57 males and 43 females. Immunopositive Her-2 expression was observed in 21% of patients, AR in 13%, ER in 3% and 0% for PR. Patients with betel quid/areca nut mouth habits had significantly absent Her-2 expression (*P* = 0.035). Also, Her-2 negative patients were also negative for AR expression (*P* = 0.002). Her-2 positive patients had poor 10-year survival (*P* = 0.041). A trend of low survival and high recurrence rate was observed in AR positive patients, but this was not significant (*P* = 0.072). No statistically relevant correlations were seen in the case of ER and PR. In conclusion, Her-2 may be a valuable marker for predicting long-term prognosis of OSCC patients.

## Introduction

Oral cancer is a global health issue due to its severe morbidity and high mortality. More than one-third of the worldwide incidence of oral cancer is found in South-Central Asia and Pakistan suffers from the second-highest rate of oral cancers worldwide [1]. Etiologic factors for oral cancer include mouth habits such as smoking, chewing tobacco, naswar, betel quid and areca nut (paan) and alcohol [2]. About 90% of oral cancers originate from the squamous epithelium of the oral cavity and are called oral squamous cell carcinomas (OSCC).

Several types of receptors have long held importance for the early detection and treatment of different cancers [3]. Their utility in targeted therapy is highlighted in the case of breast and prostate cancers, where anti-hormone agents Tamoxifen, Trastuzumab and Fulvestrant are now clinically incorporated [4, 5]. However, in the case of OSCC there is disparity in the research results regarding Her-2 and hormone receptors expression and their effect on prognosis.

Her-2 (human epidermal growth factor receptor 2) is a proto-oncogene with intrinsic tyrosine kinase activity. Her-2 regulates cell growth and differentiation in normal conditions [6]. Deregulated Her-2 can lead to uncontrolled cell division, evasion from apoptotic signals, chemoresistance, invasiveness and angiogenesis [7]. Overexpression of Her-2 leads to poor outcomes in breast, ovarian, gastric, salivary, endometrial and osteosarcoma cancers [8–10]. Concerning OSCC, some studies have suggested Her-2 as a potential target for treatment while other have contradicted any such role of Her-2 [11–13]. The efficacy of Trastuzumab, a Her-2 targeted monoclonal antibody, has been well-documented in gastric, breast and lung carcinomas, but not in OSCC [7].

On the other hand, androgen receptor (AR) is a male sex-related hormone receptor. In addition to normal prostate and mammary glands [14], it is also expressed in oral mucosa [15]. AR is thought to promote cell migration and invasiveness in cancer development [16]. AR is considered a favorable prognostic marker in head and neck squamous cell carcinoma (HNSCC) [17], while other authors have reported adverse survival in salivary gland tumors [18].

Moreover, estrogen receptor (ER) is a DNA-binding transcription factor, expressed in normal oral mucosa and salivary glands [19]. ER is divided into two subtypes: ERα and Erβ. ER dysregulation has been associated with breast, prostate, pleural mesothelioma, renal cell carcinoma, colorectal and lung cancer [20]. Increased ERα expression has been suggested to promote formation of oral tumors as well [21].

Furthermore, progesterone receptor (PR) is a nuclear transcription factor that modulates multiple organs of the reproductive system [22]. Overexpression of PR has been reported in breast, rectal and prostate cancer patients and is also believed to predispose patients to developing OSCC [23, 24]. Contrarily, increased PR expression has been noted to improve survival of HNSCC patients [25].

Thus, the objective of this study was to examine the expression of hormonal receptors, AR, PR and ER, and growth factor receptor Her-2, in OSCC and to determine their effect on patient clinicopathologic parameters,10-year survival and recurrence.

## Materials and methods

### Study site and participants

This study was conducted at Aga Khan University Hospital, Karachi which is the largest JCIA and CAP accredited tertiary-care academic medical center in Pakistan. Since it serves as the preferred referral center for all cancer cases, the resulting patient pool is diverse and well-representative of the entire population. Surgical samples from 100 untreated OSCC patients, diagnosed and treated between January 2001 and December 2010, were retrospectively collected. The availability of written informed consent, complete follow-up data, medical records and adequate tumor tissue were determined. Overall survival (OS) (minimum follow up 60 months) and 10-year survival (minimum follow up 120 months) was taken as number of months from diagnosis until last follow-up (if alive) or date of death (if dead). Disease-free survival (DFS) was calculated as the number of months starting from surgery until date of recurrence or if no recurrence then until last follow-up (if alive) or date of death. The study was approved by the institutional Ethical Review Committee (ERC#5104-Sur-ERC-17).

### Immunohistochemistry

Surgically resected primary OSCC tissue specimens in the form of formalin-fixed paraffin-embedded (FFPE) blocks were used. Diagnosis of OSCC was confirmed on H&E slides and the blocks with maximum tumor material were selected for immunohistochemistry. Sections were sliced on a semi-automatic microtome (pfm Rotary 3005 E, pfm medical, Germany) at a thickness of 4μm and transferred to pre-coated glass slides (FLEX IHC Microscope Slides, K8020, Dako). Slides were deparaffinized by heating in an oven at 56°C for 30min and dipping in xylene for 2mins. Rehydration of specimens was done using graded ethanol-water serial dilutions starting from 100% to 90%, 70% and finally 50%, followed by rinsing in distilled water.

For IHC, EnVision FLEX, High pH (Link) visualization system (K80002221, Dako) was used according to the instructions. Heat-induced epitope retrieval at high pH was done for all antibodies by immersing slides in high pH target retrieval solution (K8004, Dako) for 30 mins and placed in a water bath heated to 90–95°C. Endogenous peroxidase activity was blocked by applying peroxidase blocking reagent (S2023, Dako). A Tris buffer saline + Tween 20 combination (Wash buffer, S3006 Dako) was used for washing slides in between all steps. All anti-human primary antibodies were procured from Dako, Denmark. PR (mouse monoclonal, clone PgR 636, RRID: AB_2890066) and ERα (rabbit monoclonal, clone EP1, RRID: AB_2617140) were ready-to-use, while AR (mouse monoclonal, clone AR441, RRID: AB_2060174) was diluted 1:70 and Her-2 (rabbit polyclonal, clone A0485, RRID: AB_2335701) was diluted 1:300. Incubation time was 30 mins for all primary antibodies after which, slides were washed with wash buffer and secondary antibody EnVision/HRP (labelled-polymer Rabbit/Mouse, Dako) was applied for another 30 mins. For visualization of the antigen-antibody complex, the DAB+ Chromogen (Dako) was used for 4 mins. Slides were counterstained by dipping in hematoxylin (CS70030, Dako) and then dehydrated in a reverse series of ethanol-water dilutions starting from 50% to 70%, 90% and 100%. Toluene-free Mounting Medium (Dako) was used for mounting slides. Controls were included in each experiment. Known positive breast carcinoma was used as positive control for all antibodies, while saline in place of primary antibody served as negative control.

### Evaluation of slides

Two observers (SMAA and RI) independently reviewed the slides on a light microscope, blinded to the patient information. In case of a discrepancy, a conference microscope was used to come to a census. Approximately 200 cells each in 8–10 different fields were observed under 20x before assigning a score. The first field selected was subjective, while the remaining fields were selected methodically to assess the specimen thoroughly. AR, ER and PR display nuclear staining, while Her-2 expression is membranous. For AR and ER, positive cells were scored according to the intensity and percentage of cells stained: no cells stained = negative (0); ≤10% of cells stained = mild (+1); >10% - 50% of cell stained = moderate (+2) and ≥50% of cells stained = strong (+3) [26]. In the case of PR, no stained cells were negative and >1 weakly staining cells were positive [27]. Her-2 scoring evaluation was performed according to the American Society of Clinical Oncology/College of American Pathologists (ASCO/CAP) guidelines summarized as follows: 0 = no staining or membrane staining in <10% of tumor cells; +1 = faint staining in >10% of tumor cells and membrane partly stained; +2 weak to moderate complete membrane staining in >10% of tumor cells; +3 strong complete membrane staining in >30% of cells [28].

### Statistical analysis

The SPSS version 19 (IBM, USA) was used for statistical analysis. Cross-tabulations were run to correlate the expression of AR, PR, ER and Her-2 with patient parameters and significance was checked using Chi-Square or Fisher’s Exact test. Kaplan-Meier survival curves were drawn for 10-year and DFS using Log-rank statistic. Univariate cox regression analysis was also used to evaluate the effect of markers expression and other parameters on 10-year, OS and DFS. Hazard ratios (HR) were reported with 95% confidence interval and all *P* values were 2-sided and considered statistically significant at <0.05.

## Results

### Patient characteristics

A total of 100 patients between the ages of 20 and 78 years with a median age of 50 years (mean = 51.42, SD± 13.33) were part of this study. Eighteen patients were <40 years old and 82 patients were ≥ 40 years; while 57 patients were male and 43 were female, forming a male-female ratio of 1.33:1. 79% of patients had habits of smoking, chewing tobacco or betel nut/areca nut, while 21% of patients had no mouth habits.

The cheek was the primary site of lesion in 63 patients, whereas 37 patients presented with primary lesion in the tongue, which was probably due to the regional risk factors such as smokeless tobacco and betel quid and areca nut chewing habits. With disease progression, we observed that patients developed lesions in other sites of the oral cavity such as floor of mouth, mandible, palate, tonsils, and facial skin. Some patients presented with advanced disease and already formed secondary lesions at their first clinic visit, while others developed these with the passage of time observed during follow-up clinic visits. In cases where the patients had multiple tumor sites at their first visit, while taking patient history, the patients were asked to identify where the first lesion had developed, and this was considered the site of origin (primary tumor site). The site of origin and involvement of secondary tumor sites is specified in Table 1.

**Table 1:** Clinicopathologic parameters correlated with Her-2, AR and ER expression.

Clinicopathologic Parameters	Total	HER-2			AR			ER		
		-ve	+ve	*P value*	-ve	+ve	*P value*	-ve	+ve	*P value*
**Age (Years)**<40										
≥40	18	13	5	0.436	16	2	1	80	1	0.452
	82	66	16		71	11		17	2	
**Gender**										
Male	57	44	13	0.609	50	7	0.805	55	2	1
Female	43	35	8		37	6		42	1	
**Habits**										
Yes	79	61	18	0.551	69	10	1	76	3	1
No	21	18	3		18	3		21	0	
**Betel Nut/Areca Nut**										
Yes	61	44	17	**0.035** *	53	8	0.966	58	3	0.279
No	39	35	4		34	5		39	0	
**Smoking/Tobacco Use**										
Yes	35	26	9	0.396	30	5	0.765	34	1	1
No	65	53	12		57	8		63	2	
**Primary Tumor Site**										
Cheek	63	49	14	0.695	52	11	0.124	61	2	1
Tongue	37	30	7		35	2		36	1	
**Palate**										
Yes	8	6	2	0.772	8	0	0.592	8	0	1
No	92	73	19		79	13		89	3	
**Mandible**										
Yes	26	19	7	0.389	23	3	1	25	1	1
No	74	60	14		64	10		72	2	
**Floor of the Mouth**										
Yes	7	6	1	1	6	1	1	7	0	1
No	93	73	20		81	12		90	3	
**Tonsil**										
Yes	2	1	1	0.378	2	0	1	2	0	0.802
No	98	78	20		85	13		95	3	
**Skin**										
Yes	3	2	1	0.511	3	0	1	3	0	1
No	97	77	20		84	13		94	3	
**Histology**										
Well Differentiated	37	31	6	0.574	31	6	0.543	36	1	0.982
Moderately Differentiated	59		15	0.292	53	6	0.387	57	2	0.851
Poorly Differentiated	4	44	0	0.999	3	1	0.661	4	0	0.999
		4								
**Primary Margins**										
Clear	62	50	12	0.120	53	9	0.837	59	3	1
Near	27		4	0.609	24	3	0.666	49	0	0.998
Involved	11	23	5	0.069	10	1	0.633	22	0	0.999
		6								
**T Classification**										
T1	21	18	3	0.587	18	3	0.996	21	0	1
T2	47		11	0.395	41	6	0.864	44	3	0.998
T3	15	36	2	0.935	13	2	0.935	15	0	1
T4	17		5	0.264	15	2	0.819	17	0	1
		13								
		12								
**N Classification**										
N0	77	62	15	0.306	65	12	0.765	75	2	0.666
N1	13		2	0.728	12	1	0.464	12	1	0.367
N2	10	11	4	0.152	10	0	0.999	10	0	0.999
		6								
**AJCC Clinical Stage**										
I	19	16	3	0.262	16	3	0.816	19	0	0.993
II	32		6	0.789	27	5	0.988	30	2	0.998
III	23	26	3	0.800	20	3	0.800	22	1	0.998
IV	26	20	9	0.168	24	2	0.402	26	0	1
		17								
**Radiotherapy**										
Yes	65	50	15	0.487	56	9	1	64	1	0.280
No	25	29	6		31	4		33	2	
**HER-2**										
Positive	21	-	-	-	14	7	**0.002** *	20	1	0.511
Negative	79	-	-		73	6		77	2	
**AR**										
Positive	13	6	7	**0.002** *	-	-	-	12	1	0.344
Negative	87	73	14		-	-		85	2	
**ER**										
Positive	3	2	1	0.511	2	1	0.344	-	-	-
Negative	97	77	20		85	12		-	-	
**Recurrence**										
Yes	74	62	12	**0.048** *	68	6	**0.036** *	72	2	1
No	26	17	9		19	7		25	1	
**Survival Status**										
Alive	44	33	11	0.384	35	9	**0.049** *	42	2	0.581
Dead	56	46	10		52	4		55	1	

* *P* value significant at <0.05

On histological examination, it was found that 37% of participants had a well differentiated tumor, 59% had moderately differentiated, and 4 patients had a poorly differentiated tumor. According to the American Joint Committee on Cancer (AJCC) staging, 19% were classified as Stage I, 32% were Stage II, 23% were Stage III, and 26% were at stage IV of the disease. TNM classification revealed that 21% of participants presented with tumor size T1, 47% had T2, 15% had T3, and 17% presented with T4. While 77% of patients had no lymph node involvement, 13% were classified as N1 and 10% were classified as N2. None of the 100 cases presented with any distant metastases. All patients underwent surgical resection for their tumors, and 65% underwent radiotherapy as well. Clear surgical margins were achieved in 62%, near margins in 27%, and involved margins in 11% of cases.

### Her-2 and hormonal markers expression and correlation with patient characteristics

**Fig 1:**
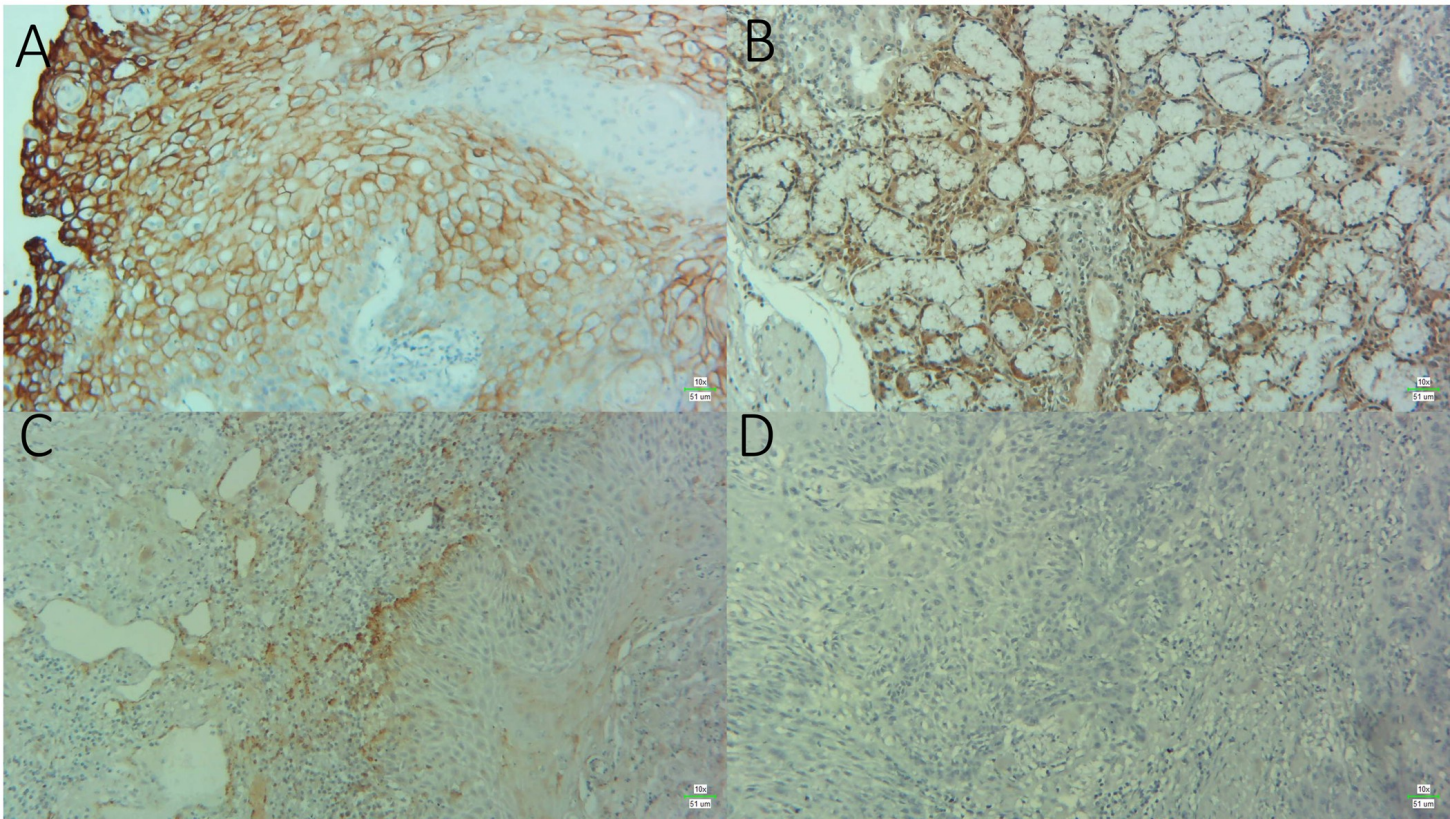
Photomicrograph of (A) Her-2 showing strong membranous positivity (B) AR showing nuclear positivity (C) ER showing nuclear positivity and (D) PR negative staining in OSCC specimens [Magnification × 10].

Her-2 expression presented as partial or complete membranous staining of tumor cells (Fig 1A) and was positive in 21% and negative in 79% of patients. Out of the 21 positive samples, 6 stained strongly, 7 stained moderately, and 8 stained mildly. It was seen that, 72% of patients with betel quid/areca nut chewing habits were markedly negative for Her-2 expression (*P* = 0.035). Another significant finding was that 84% of AR negative samples were also negative for Her-2 (*P* = 0.002), suggesting a directly proportional relationship between the two markers expression. Furthermore, 84% of patients that had recurrence were also Her-2 negative (*P* = 0.048), see Table 1 for details.

AR was positively expressed in the tumor cell nuclei (Fig 1B) of 13 patients and negative in 87. Out of the 13 positive samples, 5 were mild, 7 were moderate and 1 was strongly positive. Moreover, 92% of patients that suffered recurrence were AR negative (*P* = 0.036) and 93% of patients that had died in the follow-up duration were also AR negative (*P* = 0.049). As stated above, AR negative expression was also correlated with Her-2 negative expression status (*P* = 0.002). Table 1 lists complete correlations.

Nuclear ER expression (Fig 1C) was observed in 3 patients while it was absent in 97. All 3 specimens had mild positivity as defined by the staining criteria. ER was not found significantly associated with any patient parameter or markers expression, detailed in Table 1.

In the case of PR, no positive expression was seen in any of the tested specimens (Fig 1D). A noteworthy observation was that cytoplasmic positivity was present in several cases for PR and ER both. But this was disregarded as non-specific and only clear nuclear signals were taken as positive, according to the staining criteria.

### Overall survival (OS)

The OS rate for our cohort was 44%, and the median survival months were 64 on the Kaplan-Meier analysis. Several factors decreased the overall survival of patients (Table 2). The involvement of tonsils, as a secondary tumor site, severely reduced OS (*P*<0.001) so did metastatic lymph nodes (*P* = 0.001), higher N stage (*P*<0.001), involved surgical margins (*P* = 0.004) and advanced AJCC stage (*P* = 0.002). Receiving radiotherapy helped improve the odds of survival (*P* = 0.026). Secondary tumor sites such as palate, skin, mandible, and floor of mouth did not affect OS.

**Table 2:** Association of clinicopathologic variables and Her-2, AR and ER with overall and disease-free survival.

VARIABLE	Total	Overall Survival months	*P* Value	95% CI		Disease—free Survival months	*P* Value	95% CI	
		Median		Lower	Upper	Median		Lower	Upper
**GENDER**	** **								
MALE	57	100	0.597	37.7	162.3	51	0.629	28.1	73.9
FEMALE	43	85		30.1	139.4	58		35.4	80.6
**AGE DIVISION**									
<40 YEARS	18	155	0.93	†	†	31	0.376	0	80.9
>40 YEARS	82	100		60.2	139.8	52		35.1	69
**PRIMARY TUMOUR SITE**	** **								
CHEEK	63	85	0.287	46.6	123.4	44	**0.045** *	21.6	66.4
TONGUE	37	155		†	†	58		20.1	95.8
**TONSIL**	** **								
YES	2	9	**<0.001** *	†	†	5	**0.001** *		
NO	98	100		53.1	146.9	53		40.8	65.2
**SKIN**	** **								
YES	3	25	0.082	4.2	45.8	7	**0.031** *	2.2	11.9
NO	97	104		54.6	153.5	53		37.1	68.9
**NECK PATHOLOGY**									
POSITIVE	27	31	**0.001** *	0	102.2	69	**0.018** *	51.8	86.2
NEGATIVE	51	155		†	†	22		0	52.5
ND	22	64		13	115	27		4	50
**PATHOLOGICALLY INVOLVED LYMPH NODES**									
SINGLE	16	59	**0.001** *	2.2	115.8	29	0.078	0	85.8
MULTIPLE	11	12		6.6	17.4	6		2.8	9.2
NA	73	149		85.4	212.6	58		40.9	75.1
**PRIMARY MARGINS**									
CLEAR	62	149	**0.004** *	88.5	209.5	62	**0.008** *	49	75
NEAR	27	62		23.2	100.8	24		0	76.6
INVOLVED	11	13		9.8	16.2	7		0	15.6
**N CLASSIFICATION**									
N0	77	149	**<0.001** *	79.2	218.8	58	**0.024** *	48.6	67.4
N1	13	31		11.6	50.4	27		5.9	48.1
N2	10	12		9	15	6		2.9	9.1
**AJCC STAGE**									
I	19	249	**0.002** *	34.7	463.3	69	**0.03** *	51.8	86.3
II	32	149		82.5	215.5	58		39.7	76.3
III	23	68		47.5	88.5	51		22.8	79.2
IV	26	14		0	30.2	9		2	16
**RADIOTHERAPY**	** **								
YES	65	68	**0.026** *	43.5	92.5	44	0.242	18.3	69.7
NO	35	†		†	†	62		35	89
								35	69
**AR**									
POSITIVE	13	†	0.174	†	†	122	0.072		
NEGATIVE	87	85		44.1	126	45		15.6	74.5
**ER**									
POSITIVE	3	†	0.55	†	†	122	0.611		
NEGATIVE	97	100		61.1	139	52		35.4	68.6
**HER2**									
POSITIVE	21	68	0.753	†	†	77	0.489	26.4	127.6
NEGATIVE	79	100		52.3	147.7	52		28	76

* *P* value significant at <0.05

† statistics could not be computed

Although there was a difference in the survival months of Her-2 positive and negative patients (68 months vs 100 months respectively), this difference was not statistically valid. Similarly, AR and ER expression did not significantly differentiate between patient survival.

In the univariate OS analysis by cox regression, conventional prognostic factors were significant hazards for OS: involvement of tonsils (*P* = 0.004) involved primary margins (*P* = 0.003), T4 stage (*P* = 0.027), higher N stage (*P*<0.001), advanced AJCC stage (*P* = 0.002) and not receiving radiotherapy (*P* = 0.029). Her-2, AR, PR, and ER did not present a significant hazard for survival in our dataset. Table 3 presents cox regression univariate OS analysis.

**Table 3:** Cox regression univariate survival analysis.

Characteristic	Overall survival				Disease free survial			
	*P* value	Hazard ratio	95% CI		*P* value	Hazard ratio	95% CI	
		HR	lower	upper		HR	lower	upper
**GENDER**		100				100		
MALE		1.0(ref)				1.0(ref)		
FEMALE	0.598	1.154	0.678	1.962	0.632	0.893	0.562	1.419
**AGE DIVISION**		100				100		
< 40 YEARS		1.0(ref)				1.0(ref)		
> 40 YEARS	0.931	0.969	0.472	1.987	0.381	0.769	0.427	1.385
**PRIMARY TUMOUR SITE**		100				100		
TONGUE		1.0(ref)				1.0(ref)		
CHEEK	0.291	0.738	0.419	1.297	**0.049** *	1.655	1.002	2.734
**TONSIL**		100				100		
NO		1.0(ref)				1.0(ref)		
YES	**0.004** *	8.999	2.016	40.167	**0.007** *	7.691	1.731	34.18
**SKIN**		100				100		
NO		1.0(ref)				1.0(ref)		
YES	0.097	2.699	0.836	8.718	**0.045** *	3.316	1.029	10.69
**PRIMARY MARGINS**		100				100		
CLEAR	**0.007** *	1.0(ref)			0.11	1.0(ref)		
NEAR	**0.045** *	1.856	1.014	3.396	0.109	1.535	0.909	2.594
INVOLVED	**0.003** *	3.091	1.454	6.572	**0.004** *	2.756	1.372	5.537
**T CLASSIFICATION**		100				100		
T1	0.135	1.0(ref)			0.156	1.0(ref)		
T2	0.215	1.655	0.746	3.675	0.39	1.324	0.698	2.512
T3	0.085	2.23	0.896	5.548	0.162	1.732	0.802	3.743
T4	**0.027** *	2.803	1.124	6.991	**0.034** *	2.28	1.066	4.874
**N CLASSIFICATION**		100				100		
N0	**<0.001** *	1.0(ref)			**0.031** *	1.0(ref)		
N1	**0.004** *	2.888	1.415	5.89	0.082	1.833	0.925	3.635
N2	**<0.001** *	4.148	1.89	9.103	**0.025** *	2.364	1.114	5.018
**AJCC STAGE**		100				100		
I	**0.004** *	1.0(ref)			**0.038** *	1.0(ref)		
II	0.345	1.579	0.612	4.075	0.436	1.329	0.65	2.717
III	**0.033** *	2.789	1.088	7.129	0.143	1.755	0.827	3.726
IV	**0.002** *	4.259	1.681	10.792	**0.009** *	2.644	1.269	5.512
**RADIOTHERAPY**		100				100		
YES		1.0(ref)				1.0(ref)		
NO	**0.029** *	1.950	1.071	3.552	0.247	0.749	0.459	1.222
**AR**		100				100		
NEGATIVE		1.0(ref)				1.0(ref)		
POSITIVE	0.184	0.5	0.179	1.392	0.082	0.475	0.205	1.099
**ER**		100				100		
NEGATIVE		1.0(ref)				1.0(ref)		
POSITIVE	0.557	0.553	0.076	4.001	0.615	0.697	0.171	2.848
**HER2**		100				100		
NEGATIVE		1.0(ref)				1.0(ref)		
POSITIVE	0.754	1.117	0.559	2.233	0.494	0.805	0.432	1.499
**RECURRENCE**		100						
NO		1.0(ref)						
YES	**0.003** *	38.261	3.546	412.78	-	-	-	-

* *P* value significant at <0.05

### Disease free survival (DFS)

A total of 74 study participants had disease recurrence within 60 months of surgery and median DFS months were 52.5 on the Kaplan-Meier analysis. According to the primary tumor site, cheek patients suffered recurrence at median 44 months which was significantly earlier than tongue patients who recurred at 58 months (*P* = 0.045). Several other parameters predicted early recurrence, including tonsil involvement, as a secondary tumor site, (*P* = 0.001), N2 stage (*P* = 0.024), metastatic lymph nodes (*P* = 0.018), skin involvement, as a secondary tumor site, (*P* = 0.031), AJCC stage IV (*P* = 0.03) and involved surgical margins. (*P* = 0.008). Other secondary tumor sites such as palate, mandible and floor of mouth were insignificant factors for determining DFS.

Based on biomarker expression, AR positive patients had DFS of 122 months while negative patients had shorter DFS of 45 months, but this was borderline significant (*P* = 0.072). Moreover, no significant differences in DFS based on Her-2 and ER expression were seen. For details see Table 2.

In the univariate cox regression DFS analysis the following factors were predictors for higher odds of recurrence: cheek as primary tumor site (*P* = 0.049), involved primary margins (*P* = 0.004) tonsils involvement (*P* = 0.007), higher N stage (*P* = 0.025), higher T stage (*P* = 0.027), advanced AJCC stage (*P* = 0.009) and skin involvement (*P* = 0.045). Her-2, AR, PR and ER did not significantly increase the risk of recurrence in our patients (Table 3).

### 10-year survival

Of the 100 participants, 75 had minimum 120 months follow-up data and were selected for 10-year Kaplan-Meier survival analysis. There were only 8 patients who had survived 10 years without recurrence, while 67 had recurrence and 50 of these patients died within the follow-up duration.

Important classic predictors of outcome were significantly associated with 10-year survival. Involved tonsils (*P* = 0.004), positive neck pathology (*P* = 0.002), multiple metastatic lymph nodes (*P*<0.001), involved primary tumor margins (*P* = 0.014) absence of radiotherapy (*P* = 0.245), advanced T4 stage (*P* = 0.065), N2 stage (*P*<0.001) and AJCC stage IV (*P*<0.001) all were predictors of poor 10-year survival.

**Fig 2:**
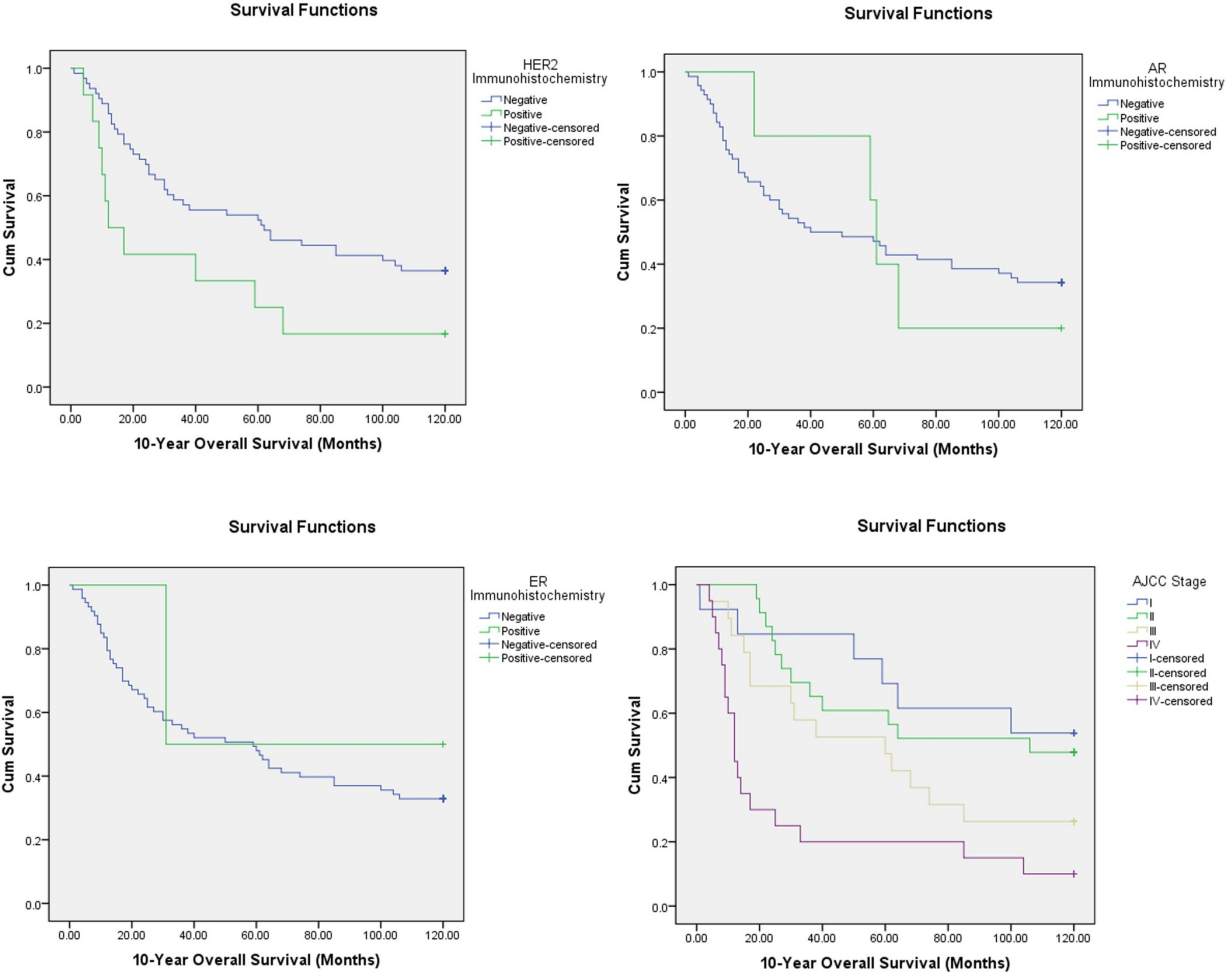
Kaplan-Meier curve analysis for 10-year survival showing Her-2 positive (*P* = 0.041) and higher AJCC stage (*P*<0.001) patients with markedly lower survival. There were no significant differences in survival on the basis of AR (*P* = 0.913) and ER (*P* = 0.615) expression.

Among the biomarkers tested, Her-2 positive patients had a significantly shorter 10-year median survival (12 months) as compared to Her-2 negative patients (62 months, *P* = 0.041), see Fig 2. AR and ER status had no significant effect on 10-year survival in this cohort (*P* = 0.913 and *P* = 0.615 respectively).

#### Clinical correlations of Her-2 strong positive cases

Since strong membranous Her-2 positivity has several clinical implications, such as predicting response to therapy, the staining score of Her-2 cases (negative, mild, moderate, or strong) was correlated with patient clinicopathologic characteristics and survival to delineate the impact of strong Her-2 positivity. Among the Her-2 positive patients, 6 stained strongly, out of which 3 were male and female each. Most of the Her-2 strong positive patients were >40 years of age (5/6) and had chewing habits (5/6) with betel quid/areca nut as the most popular substance (5/6).

The cheek was the predominant primary tumor site for Her-2 strong positive cases (5/6) which may be explained by the prevalent chewing habits with moderately differentiated tumors in 4/6 and well-differentiated tumors in 2/6. Interestingly, none of the patients had tonsils, facial skin, or floor of the mouth as secondary tumor sites. The AJCC stage was IV in 3 cases, II in 2 cases and III in 1 case. Recurrence was observed in 4/6 patients and 3/6 patients had expired in the follow-up duration.

As determined by Kaplan-Meier analysis, the OS of strong Her-2 positive patients was significantly lower at median 40 months vs. 100 months for Her-2 negative patients (*P* = 0.022). Additionally, the 10-year survival of Her-2 strong positive patients was reduced to a median of 10 months, as compared to 62 months of survival of Her-2 negative patients (*P* = 0.003). While the DFS was also lower for Her-2 strong positive cases at median 21 months as compared to 52 months for Her-2 negative patients, this did not reach statistical significance (*P* = 0.092).

## Discussion

OSCC has one of the lowest 5-year survival rates, remaining below 50% for the last three decades [29]. While emphasis has been placed on 5-year survival, there are very few studies reporting the 10-year survival of OSCC patients. Long-term survival analysis allows the assessment of several clinicopathologic parameters over the period of time, and to evaluate their changing influence on patient mortality. A single-centre study of China has reported a decrease in survival rate from 71% at 3-year, to 64% at 5-year and 54% at 10-years for OSCC [30].

This study reports reduced 10-year survival for OSCC that are Her-2 positive. Existing literature has emphasized more on the expression of Her-2 in HNSCC, as compared to OSCC alone. The immunopositivity of Her-2 has ranged from 0% - 40% in HNSCC [31, 32], and from 2.5% upto 90% in OSCC [33]. The disparities in Her-2 positivity may be due to differences in staining and scoring protocols. Some authors have considered cytoplasmic Her-2 expression, while others argue that cytoplasmic staining is non-specific and consider only membranous signals [33]. Likewise, we followed the ASCO/CAP established criteria for scoring Her-2 expression and only considered membranous signals as positive.

Many studies have reported no Her-2 expression, while others have found no worthy correlations in OSCC [32, 34–36]. In other works, however, overexpression of Her-2 has been associated with significantly shorter overall survival, higher clinical and nodal stage, and metastasis of OSCC patients [37–40]. Similarly, we found shorter overall survival in Her-2 positive vs negative patients. Also, the rate of recurrence was lower in Her-2 positive patients. This may be due to the large number of Her-2 negative patients who might have suffered recurrence due to other factors such as expression of other oncogenic proteins, continued use of risk factors, not receiving, or completing radiotherapy etc. Usually, the effect of Her-2 on recurrence has been observed solely on Her-2 positive cohorts to provide a better understanding [41].

Interestingly, Her-2 was significantly negative in patients who had betel quid/areca nut chewing habits as 72% of chewers were Her-2 negative (*P* = 0.035). Chewing areca nut (either alone or in combination with betel quid) is the 4^th^ most common mouth habit in the world. The main alkaloid in areca nut is arecoline, known to cause oral potentially malignant disorders and promote cancer. An in-vitro study on endometrial cells also found that arecoline downregulated the expression of Her-2 protein [42]. This may be the case in our patients, as well as continuous areca nut use might have encouraged the development of Her-2 negative tumors. Further case-control studies may elucidate the action of Her-2 in chewers and non-chewers and the effects on patient outcomes.

Trastuzumab is a monoclonal antibody that binds to and blocks Her-2 functioning and encourages the immune system attack against the tumor. Her-2 positive breast cancer patients traditionally respond well to Trastuzumab and face improved OS, decreased risk of recurrence and metastasis after treatment [43]. Promising anti-tumor effects of Trastuzumab have been observed in-vitro in combination with gefitinib or cetuximab in HNSCC patients [44]. Hence, as part of an OSCC clinical trial, Her-2 overexpression can be used to identify a sub-set of OSCC patients who may respond well to anti-Her-2 therapy and potentially improve their survival.

In the case of AR, the immunohistochemical expression has ranged from 36% in HNSCC [17] to 67% in OSCC [45]. AR has also been considered as a reliable predictor for metastasis risk in OSCC patients [46], and deemed critical for maintaining oral malignancy and continued cell growth [45]. However, Mohamed et al. [26] found AR ineffectual for survival prediction in oropharyngeal cancers. In our cohort of OSCC patients, there was a trend of shorter survival and higher recurrence rate for AR negative patients, but this was borderline significant on Kaplan-Meier analysis (*P* = 0.072). Further molecular investigations into the mechanism by which AR is downregulated may help understand the effects on disease progression.

Limited studies have evaluated the role of ER in OSCC. ER exists as ERα and ERβ and expression varies according to the subtype and tissue under study. Previous studies suggest that tongue and oropharyngeal cancers express ERβ and not Erα [26, 47]. But, even in the case of absent ERα protein expression, ERα mRNA has been detected in cervical carcinoma and this suggests a post-transcriptional regulation of ERα, which may be true in the case of OSCC as well [48].

A study by Vaalima et al. [19] tested oral mucosa and salivary gland tissues for the presence of both ER subtypes and found high expression of ERβ in both tissues, and no ERα expression. Though we found only 3 positive cases for ERα, a previous study on OSCC found 11% immunopositivity [24]. Furthermore, ER positive laryngeal and hypolaryngeal patients have been found to have reduced OS but this has not been true for oral cavity cancers [49]. Although the median disease-free survival months for our patients were greater for ER positive vs ER negative, these differences were not significant, plausibly due to the low number of positive cases. Even so, the subset of ER positive OSCC patients may benefit from tamoxifen therapy, which is a known ER modulator used in the treatment of breast cancer. Treatment with Tamoxifen has shown inhibition of cell proliferation and invasion in in-vitro studies on OSCC cell lines [24].

Although there are very few comparative studies available, in this study group, all samples were negative for PR nuclear expression. This is consistent with an earlier study reporting no PR expression in normal oral mucosa, oral hyperplasia, squamous intraepithelial neoplasia and OSCC [24]. Contrarily, other investigations have found up to 40% PR positivity in oropharyngeal cancers which is considered a distinct site to oral cavity cancers [26]. However, the authors also considered the cytoplasmic expression of PR as positive, as opposed to only nuclear expression. We noted cytoplasmic PR expression in approximately 30% of cases and this was discussed in the Pathology Department Consultant Conference. The consensus was that since PR was a nuclear receptor, cytoplasmic staining didn’t represent functional PR protein expression. This is in concordance with other studies validating several PR antibodies (including the PgR636 clone used in this study), and only considering nuclear positivity[27, 50]. Consequently, PR overexpression does not seem to play a role in OSCC.

Future clinical trials may be designed to explore the treatment options for patients that are hormone-receptor or Her-2 positive as demonstrated here, can benefit from targeted therapy. The present study adds unique insights to our current understanding of oral cancer and hormonal receptors and Her-2 using a well-characterized cohort from a high-risk population.

## Conclusion

Her-2 positivity significantly predicts poor long-term prognosis in OSCC patients. Her-2 positive OSCC patients may be good candidates for treatment with Trastuzumab. Similarly, AR+ patients may be treated with androgen-deprivation therapy to improve survival and decrease chances of recurrence. No role of ER or PR expression in OSCC could be determined in this study. Important classic predictors of survival such as AJCC and TNM staging were consistently associated with poor patient survival.

## References

[1] Sung H, Ferlay J, Siegel RL, Laversanne M, Soerjomataram I, Jemal A, et al. Global cancer statistics 2020: GLOBOCAN estimates of incidence and mortality worldwide for 36 cancers in 185 countries. CA Cancer J Clin. 2021;71(3):209–49. doi: 10.3322/caac.21660 33538338

[2] Irani S. New insights into oral cancer—Risk factors and prevention: A review of literature. Int J Prev Med. 2020;11. doi: 10.4103/ijpvm.IJPVM_320_18 33815726 PMC8000242

[3] Henderson BE, Feigelson HS. Hormonal carcinogenesis. Carcinogenesis. 2000;21(3):427–33. doi: 10.1093/carcin/21.3.427 10688862

[4] Narod SA, Sopik V, Sun P. Which women decide to take tamoxifen? Breast Cancer Res Treat. 2017;164(1):149–55. doi: 10.1007/s10549-017-4226-4 28374324

[5] Gasent Blesa J, Alberola Candel V, Giner Marco V, Giner-Bosch V, Provencio Pulla M, Laforga Canales J. Experience with fulvestrant acetate in castration-resistant prostate cancer patients. Ann Oncol. 2010;21(5):1131–2. doi: 10.1093/annonc/mdq010 20139153

[6] Seifi S, Shafaei S, Nosrati K, Ariaeifar B. Lack of elevated HER2/neu expression in epithelial dysplasia and oral squamous cell carcinoma in Iran. Asian Pac J Cancer Prev. 2009;10(4):661–4. 19827890

[7] Mirza S, Hadi N, Pervaiz S, Khan SZ, Mokeem SA, Abduljabbar T, et al. Expression of HER-2/neu in oral squamous cell carcinoma. Asian Pac J Cancer Prev. 2020;21(5):1465. doi: 10.31557/APJCP.2020.21.5.1465 32458657 PMC7541867

[8] Bonello M, Sims AH, Langdon SP. Human epidermal growth factor receptor targeted inhibitors for the treatment of ovarian cancer. Cancer Biol Med. 2018;15(4):375. doi: 10.20892/j.issn.2095-3941.2018.0062 30766749 PMC6372909

[9] Khan AJ, King BL, Smith BD, Smith GL, DiGiovanna MP, Carter D, et al. Characterization of the HER-2/neu oncogene by immunohistochemical and fluorescence in situ hybridization analysis in oral and oropharyngeal squamous cell carcinoma. Clin Cancer Res. 2002;8(2):540–8. 11839675

[10] Omar N, Yan B, Salto-Tellez M. HER2: An emerging biomarker in non-breast and non-gastric cancers. Pathogenesis. 2015;2(3):1–9.

[11] Dragomir L, Mărgăritescu C, Florescu A, Olimid A, Dragomir M, Popescu M. The immunoexpression of EGFR and Her2/neu in oral squamous carcinoma. Rom J Morphol Embryol. 2012;53(3):597–601. 22990553

[12] Kouhsoltani M, Aghbali A, Shokoohi B, Ahmadzadeh R. Molecular targeting of Her-2/neu protein is not recommended as an adjuvant therapy in oral squamous cell carcinoma and oral lichen planus. Adv Pharm Bull. 2015;5(Suppl 1):649. doi: 10.15171/apb.2015.088 26793611 PMC4708036

[13] Schartinger V, Schmutzhard J, Wurm M, Schwentner I, Obrist P, Oberaigner W, et al. The expression of EGFR, HER2 and EpCAM in head and neck squamous cell carcinomas. Memo. 2009;2(1):45–50.

[14] Ruizeveld de Winter J, Trapman J, Vermey M, Mulder E, Zegers ND, van der Kwast TH. Androgen receptor expression in human tissues: an immunohistochemical study. J Histochem Cytochem. 1991;39(7):927–36. doi: 10.1177/39.7.1865110 1865110

[15] Ojanotko-Harri A, Forssell H, Laine M, Hurttia H, Bläuer M, Tuohimaa P. Immunohistochemical detection of androgen receptors in human oral mucosa. Arch Oral Biol. 1992;37(6):511–4. doi: 10.1016/0003-9969(92)90108-k 1637265

[16] Zhang Y, Pan T, Zhong X, Cheng C. Androgen receptor promotes esophageal cancer cell migration and proliferation via matrix metalloproteinase 2. Tumour Biol. 2015;36(8):5859–64. doi: 10.1007/s13277-015-3257-x 25724186

[17] Rades D, Seibold N, Schild S, Gebhard M, Noack F. Androgen receptor expression. Strahlenther Onkol. 2013;189(10):849–55. doi: 10.1007/s00066-013-0389-z 23959264

[18] Luk PP, Weston JD, Yu B, Selinger CI, Ekmejian R, Eviston TJ, et al. Salivary duct carcinoma: clinicopathologic features, morphologic spectrum, and somatic mutations. Head Neck. 2016;38(S1):E1838–E47. 26699379 10.1002/hed.24332

[19] Valimaa H, Savolainen S, Soukka T, Silvoniemi P, Makela S, Kujari H, et al. Estrogen receptor-beta is the predominant estrogen receptor subtype in human oral epithelium and salivary glands. J Endocrinol. 2004;180(1):55–62. doi: 10.1677/joe.0.1800055 14709144

[20] Paterni I, Granchi C, Katzenellenbogen JA, Minutolo F. Estrogen receptors alpha (ERα) and beta (ERβ): subtype-selective ligands and clinical potential. Steroids. 2014;90:13–29. Epub 2014/06/24. doi: 10.1016/j.steroids.2014.06.012 24971815 PMC4192010

[21] Colella G, Izzo G, Carinci F, Campisi G, Lo Muzio L, D’Amato S, et al. Expression of sexual hormones receptors in oral squamous cell carcinoma. Int J Immunopathol Pharmacol. 2011;24(2_suppl):129–32. doi: 10.1177/03946320110240S222 21781458

[22] Yip CH, Rhodes A. Estrogen and progesterone receptors in breast cancer. Future Oncol. 2014;10(14):2293–301. Epub 2014/12/05. doi: 10.2217/fon.14.110 . 25471040

[23] Boccellino M, Di Stasio D, Dipalma G, Cantore S, Ambrosio P, Coppola M, et al. Steroids and growth factors in oral squamous cell carcinoma: useful source of dental-derived stem cells to develop a steroidogenic model in new clinical strategies. Eur Rev Med Pharmacol Sci. 2019;23(20):8730–40. Epub 2019/11/07. doi: 10.26355/eurrev_201910_19267 . 31696459

[24] Grimm M, Biegner T, Teriete P, Hoefert S, Krimmel M, Munz A, et al. Estrogen and progesterone hormone receptor expression in oral cavity cancer. Med Oral Patol Oral Cir Bucal. 2016;21(5):e554. doi: 10.4317/medoral.21182 27475696 PMC5005091

[25] Berg N, Neel H, Weiland L, editors. Progesterone receptors in carcinomas of the upper aerodigestive tract and lung: Biochemistry and clinical significance. Surg Forum; 1987. 10.1177/019459988910100503 2512530

[26] Mohamed H, Aro K, Jouhi L, Mäkitie A, Remes S, Haglund C, et al. Expression of hormone receptors in oropharyngeal squamous cell carcinoma. European archives of oto-rhino-laryngology: official journal of the European Federation of Oto-Rhino-Laryngological Societies (EUFOS): affiliated with the German Society for Oto-Rhino-Laryngology—Head and Neck Surgery. 2018;275:1289–300. doi: 10.1007/s00405-018-4949-9 . 29582173

[27] Mohsin SK, Weiss H, Havighurst T, Clark GM, Berardo M, Roanh LD, et al. Progesterone receptor by immunohistochemistry and clinical outcome in breast cancer: a validation study. Mod Pathol. 2004;17(12):1545–54. doi: 10.1038/modpathol.3800229 15272277

[28] Wolff AC, Hammond MEH, Hicks DG, Dowsett M, McShane LM, Allison KH, et al. Recommendations for Human Epidermal Growth Factor Receptor 2 Testing in Breast Cancer: American Society of Clinical Oncology/College of American Pathologists Clinical Practice Guideline Update. J Clin Oncol. 2013;31:3997–4013. doi: 10.1200/JCO.2013.50.9984 24101045

[29] Sasahira T, Kirita T. Hallmarks of Cancer-Related Newly Prognostic Factors of Oral Squamous Cell Carcinoma. Int J Mol Sci. 2018;19:2413. doi: 10.3390/ijms19082413 30115834 PMC6121568

[30] Bai X-X, Zhang J, Wei L. Analysis of primary oral and oropharyngeal squamous cell carcinoma in inhabitants of Beijing, China—a 10-year continuous single-center study. BMC Oral Health. 2020;20(1):1–7. doi: 10.1186/s12903-020-01192-6 32680501 PMC7367409

[31] Cavalot A, Martone T, Roggero N, Brondino G, Pagano M, Cortesina G. Prognostic impact of HER-2/neu expression on squamous head and neck carcinomas. Head Neck. 2007;29:655–64. doi: 10.1002/hed.20574 . 17315173

[32] Sardari Y, Pardis S, Tadbir AA, Ashraf MJ, Fattahi MJ, Ebrahimi H, et al. HER2/neu expression in head and neck squamous cell carcinoma patients is not significantly elevated. Asian Pacific journal of cancer prevention: APJCP. 2012;13:2891–6. doi: 10.7314/apjcp.2012.13.6.2891 . 22938479

[33] Papavasileiou D, Tosios K, Christopoulos P, Goutas N, Vlachodimitropoulos D. Her-2 immunohistochemical expression in oral squamous cell carcinomas is associated with polysomy of chromosome 17, not Her-2 amplification. Head Neck Pathol. 2009;3(4):263. doi: 10.1007/s12105-009-0134-1 20596843 PMC2811573

[34] Shintani S, Nakahara Y, Li C, Mihara M, Nakashiro K-i, Hamakawa H. HER2/neu Expression in Oral Squamous Cell Carcinoma. Asian Journal of Oral and Maxillofacial Surgery. 2004;16:172–6. 10.1016/S0915-6992(04)80028-1.

[35] Ekberg T, Nestor M, Engström M, Nordgren H, Wester K, Carlsson J, et al. Expression of EGFR, HER2, HER3, and HER4 in metastatic squamous cell carcinomas of the oral cavity and base of tongue. International journal of oncology. 2005;26:1177–85. doi: 10.3892/ijo.26.5.1177 . 15809707

[36] Angiero F, Sordo RD, Dessy E, Rossi E, Berenzi A, Stefani M, et al. Comparative analysis of c-erbB-2 (HER-2/neu) in squamous cell carcinoma of the tongue: does over-expression exist? And what is its correlation with traditional diagnostic parameters? J Oral Pathol Med. 2008;37:145–50. doi: 10.1111/j.1600-0714.2007.00603.x . 18251938

[37] Xia W, Lau Y-K, Zhang H-Z, Liu A-R, Li L, Kiyokawa N, et al. Strong correlation between c-erbB-2 overexpression and overall survival of patients with oral squamous cell carcinoma. Clin Cancer Res. 1997;3(1):3–9. 9815530

[38] Xia W, Lau Y-K, Zhang H-Z, Xiao F-Y, Johnston DA, Liu A-R, et al. Combination of EGFR, HER-2/neu, and HER-3 is a stronger predictor for the outcome of oral squamous cell carcinoma than any individual family members. Clin Cancer Res. 1999;5(12):4164–74. 10632356

[39] Kuropkat C, Venkatesan T, Caldarelli D, Panje W, Hutchinson J, Preisler H, et al. Abnormalities of molecular regulators of proliferation and apoptosis in carcinoma of the oral cavity and oropharynx. Auris Nasus Larynx. 2002;29(2):165–74. doi: 10.1016/s0385-8146(01)00129-8 11893452

[40] Cierpikowski P, Lis-Nawara A, Gajdzis P, Bar J. PDGFRα/HER2 and PDGFRα/p53 Co-expression in Oral Squamous Cell Carcinoma. Anticancer Res. 2018;38:795–802. doi: 10.21873/anticanres.12286 . 29374704

[41] O’Shaughnessy J, Robert N, Annavarapu S, Zhou J, Sussell J, Cheng A, et al. Recurrence rates in patients with HER2+ breast cancer who achieved a pathological complete response after neoadjuvant pertuzumab plus trastuzumab followed by adjuvant trastuzumab: a real-world evidence study. Breast Cancer Res Treat. 2021:1–11. doi: 10.1007/s10549-021-06137-3 33649981

[42] Giri S, Poindexter KM, Sundar SN, Firestone GL. Arecoline induced disruption of expression and localization of the tight junctional protein ZO-1 is dependent on the HER 2 expression in human endometrial Ishikawa cells. BMC Cell Biol. 2010;11(1):1–12. doi: 10.1186/1471-2121-11-53 20604955 PMC2910664

[43] Patel A, Unni N, Peng Y. The changing paradigm for the treatment of HER2-positive breast cancer. Cancers (Basel). 2020;12(8):2081. doi: 10.3390/cancers12082081 32731409 PMC7464074

[44] Hanken H, Gaudin R, Gröbe A, Fraederich M, Eichhorn W, Smeets R, et al. Her2 expression and gene amplification is rarely detectable in patients with oral squamous cell carcinomas. J Oral Pathol Med. 2014;43:304–8. doi: 10.1111/jop.12173 . 24645976

[45] Wu T-F, Luo F-J, Chang Y-L, Huang C-M, Chiu W-J, Weng C-F, et al. The oncogenic role of androgen receptors in promoting the growth of oral squamous cell carcinoma cells. Oral Dis. 2015;21:320–7. doi: 10.1111/odi.12272 . 25040852

[46] Tomasovic-Loncaric C, Fucic A, Andabak A, Andabak M, Ceppi M, Bruzzone M, et al. Androgen Receptor as a Biomarker of Oral Squamous Cell Carcinoma Progression Risk. Anticancer Res. 2019;39:4285–9. doi: 10.21873/anticanres.13593 . 31366519

[47] Ishida H, Wada K, Masuda T, Okura M, Kohama K, Sano Y, et al. Critical role of estrogen receptor on anoikis and invasion of squamous cell carcinoma. Cancer science. 2007;98:636–43. doi: 10.1111/j.1349-7006.2007.00437.x . 17355262 PMC11158041

[48] López-Romero R, Garrido-Guerrero E, Rangel-López A, Manuel-Apolinar L, Piña-Sánchez P, Lazos-Ochoa M, et al. The cervical malignant cells display a down regulation of ER-α but retain the ER-β expression. International journal of clinical and experimental pathology. 2013;6:1594–602. . 23923078 PMC3726975

[49] Lukits J, Remenár E, Rásó E, Ladányi A, Kásler M, Tímár J. Molecular identification, expression and prognostic role of estrogen- and progesterone receptors in head and neck cancer. Int J Oncol. 2007;30:155–60. doi: 10.3892/ijo.30.1.155 . 17143524

[50] Press M, Spaulding B, Groshen S, Kaminsky D, Hagerty M, Sherman L, et al. Comparison of different antibodies for detection of progesterone receptor in breast cancer. Steroids. 2002;67(9):799–813. doi: 10.1016/s0039-128x(02)00039-9 12123792

